# Sequential Isolation in a Patient of *Raoultella planticola* and *Escherichia coli* Bearing a Novel IS*CR1* Element Carrying *bla*_NDM-1_

**DOI:** 10.1371/journal.pone.0089893

**Published:** 2014-03-03

**Authors:** Juan Li, Ruiting Lan, Yanwen Xiong, Changyun Ye, Min Yuan, Xinfeng Liu, Xia Chen, Deshan Yu, Bin Liu, Wenchao Lin, Xuemei Bai, Yan Wang, Qiangzheng Sun, Yiting Wang, Hongqing Zhao, Qiong Meng, Qiang Chen, Ailan Zhao, Jianguo Xu

**Affiliations:** 1 State Key Laboratory for Infectious Disease Prevention and Control, Collaborative Innovation Center for Diagnosis and Treatment of Infectious Disease, National Institute for Communicable Disease Control and Prevention, China CDC, Changping, Beijing, China; 2 School of Biotechnology and Biomolecular Sciences, University of New South Wales, Sydney, Australia; 3 Gansu Provincial Center for Disease Control and Prevention, Lanzhou, Gansu Province, China; 4 College of Life Sciences, Nankai University, Tianjin, China; University of Manchester, United Kingdom

## Abstract

**Background:**

The gene for New Delhi metallo-β-lactamase 1 (NDM-1) has been reported to be transmitted via plasmids which are easily transferable and capable of wide distribution. We report the isolation of two NDM-1 producing strains and possible *in vivo* transfer of *bla*_NDM-1_ in a patient.

**Methods:**

Clinical samples were collected for bacterial culture and antibiotic susceptibility testing from a patient during a 34-day hospitalization. The presence of *bla*_NDM-1_ was detected by PCR and sequencing. Plasmids of interest were sequenced. Medical records were reviewed for evidence of association between the administration of antibiotics and the acquisition of the NDM-1 resistance.

**Results:**

A NDM-1 positive *Raoultella planticola* was isolated from blood on the ninth day of hospitalization without administration of any carbapenem antibiotics and a NDM-1 positive *Escherichia coli* was isolated from feces on the 29^th^ day of hospitalization and eight days after imipenem administration. The *bla*_NDM-1_ was carried by a 280 kb plasmid pRpNDM1-1 in *R. planticola* and a 58 kb plasmid pEcNDM1-4 in *E. coli*. The two plasmids shared a 4812 bp NDM-1-IS*CR1* element which was found to be excisable from the plasmid as a free form and transferrable in vitro to a NDM-1 negative plasmid from *E. coli*.

**Conclusion:**

*bla*_NDM-1_ was embedded in an IS*CR1* complex class 1 integron as a novel 4812 bp NDM-1-IS*CR1* element. The element was found to be able to self excise to become a free form, which may provide a new vehicle for NDM-1 dissemination. This mechanism could greatly accelerate the spread of NDM-1 mediated broad spectrum β-lactam resistance.

## Introduction

Carbapenems often represent last-resource drugs for Gram negative bacterial infections. The most common mechanism of carbapenem resistance in Gram negative bacteria is the production of carbapenemases, including metallo-β-lactamases (MBLs). The New Delhi metallo-beta-lactamase (NDM-1), a recently identified novel type of MBL can hydrolyze virtually all β-lactams, adding further burden to an already high level of antibiotic resistance. The emergence and global spread of NDM-1 mediated broad spectrum β-lactam resistance has become a major concern worldwide [1], [2].

NDM-1 was first identified in a carbapenem-resistant *Klebsiella pneumoniae* strain isolated from urine and an *Escherichia coli* strain isolated from feces of the same patient that was resistant to almost all antibiotics tested except colistin and tigecycline in 2009 in Sweden [3]. NDM-1 positive *K. pneuomoniae* and *E. coli* were soon detected in many other countries, including Australia, Canada, Germany, China, France, Japan, the Nordic countries, United States and many others [4]–[8]. In addition, reported NDM-1 positive bacterial species extended to *Acinetobacter* spp, *Citrobacter freundii*, *Enterobacter cloacae*, *Morganella morganii*, *Providencia* spp, and *Pseudomonas aeruginosa* [9]–[11]. The world has been alarmed by the wide dissemination of NDM-1 isolates, for which new and effective antibiotics are currently unavailable. The gene *bla*_NDM-1_ encoding NDM-1 has been mostly reported to be on plasmids, which could rapidly disseminate and spread between different bacterial species by cell-to-cell transfer of the plasmids. However the plasmids carrying *bla*_NDM-1_ are different in sizes, ranging from 40 kb to 400 kb [9], suggesting that direct transfer by plasmid is only one means of NDM-1 dissemination. Multiple mobile genetic elements are also associated with *bla*_NDM-1_ dissemination [4], [11], [12].

The insertion sequence common region 1 (IS*CR1*) is a well recognized system of gene capture. Its role in antibiotic resistance dissemination was first noted by the evidence that a wide variety of antimicrobial determinants are found in the immediate vicinity of IS*CR1* [13], [14]. IS*CR1* is capable of mobilizing its upstream sequences by a process of rolling circle transposition [15]. Association of the IS*CR1* element with *bla*_NDM-1_ has been observed in plasmids pNDM-HK(HQ451074) [16], pMR0211(JN687470) [17] and in *K. pneumoniae* strain 05-506 [3] and *P. aeruginosa* [18]. However the role of the IS*CR1* element in the transfer of the NDM-1 within and between bacterial species is unclear.

Here, we report that we identified two strains of different species (*Raoultella planticola* and *E. coli*) carrying NDM-1 isolated 20 days apart from the same patient and showed that *bla*_NDM-1_ was located on two unrelated plasmids. The *bla*_NDM-1_ was embedded in an IS*CR1* complex class 1 integron, which is most likely responsible for *bla*_NDM-1_ transfer between *R. planticola* and *E. coli* in the patient in vivo.

## Materials and Methods

### Ethics statement

The feces, urine, sputum and blood samples were obtained with the written informed consent from the patient. This study was reviewed and approved by the ethics committee of the National Institute for Communicable Disease Control and Prevention, China CDC, according to the medical research regulations of the Ministry of Health, China. This research was conducted within China.

### The Patient

The patient was a 51-year-old farmer from a small village of Gansu Province, who has never visited any cities in China or other countries that have reported NDM-1. On October 1, 2010, the patient was admitted to Lanzhou medical school hospital because of severe multiple left rib fractures as a result of traffic accident. Surgical operation was performed three hours after the accident. A second operation was performed five days later on the 6^th^ of October. The patient was hospitalized for 34 days and discharged on November 6, 2010. The blood, urine, sputum and feces were sampled for isolation of bacteria and antibiotic susceptibility testing as standard patient care except that some samples also were collected for the investigation of NDM-1.

### Bacterial isolation, antibiotics resistance testing and molecular typing

Species identification was performed using the Microscan WalkAway 40 S1 identification system (Dade Behring, Deerfield, IL, USA). The 16s rDNA and *rpoB* gene sequences were also analyzed to confirm the species identity. Susceptibility of the isolates to antibiotic was determined by the E-test methodology (BioMerieux SA, La Balme-les-Grottes, France). Modified Hodge test was performed to screen for carbapenemases. The results were interpreted according to the 2011 Clinical and Laboratory Standards Institute recommendation [19]. The relationship of NDM-1 positive and negative *E. coli* strains isolated from the same fecal samples was analyzed by PFGE (Pulsed Field Gel Electrophoresis)-*Xba* I digestion method, following the protocol previously described [20].

### PCR primers and detection of *bla*_NDM-1_

PCR primers for detecting *bla*_NDM-1_ and for confirming the genetic environment of the *bla*_NDM-1_ are listed in Table S1 in File S1. Plasmids were analyzed and sized by the PFGE-S1 nuclease method [21]. Separation of large fragments from restriction enzyme digestion was done with PFGE by using of a CHEF-DR III apparatus (Bio-Rad, Hercules, CA) for 7 h at 6 V/cm and 14°C with initial and final pulse times of 5 s and 20 s, respectively. Southern hybridization was performed with a 560 bp segment of the *bla*_NDM-1_ gene as a probe using the ECL direct nucleic acid labeling and detection system (GE Healthcare, Buckinghamshire, UK).

### Conjugation and transformation of *bla*_NDM-1_ plasmids

Conjugation experiments were carried out by the solid surface methods with *E. coli* J53 Az^r^ as the recipient [4]. Transconjugants were selected on Luria-Bertani agar plates containing sodium azide (100 mg/L) and ampicillin (100 mg/L), confirmed by PCR amplification of the *bla*_NDM-1_ for NDM-1 positive donors and *bla*_OXA-30_ gene for NDM-1 negative donors and sequencing of the PCR products. The plasmid DNA of NDM-1 positive strain *E. coli* EcNDM1was used for transformation and *E. coli* JM109 was used as the recipient.

### Whole genome sequencing and sequencing of plasmids

The whole genome of the isolate *R. planticola* was sequenced using a combined strategy of Roche 454 Genome Sequencer FLX System (Roche/454 Life Sciences, Branford, CT, USA) and Illumina sequencing technology (Illumina, San Diego, CA, USA). The Burrows-Wheeler Alignment (BWA) tool was used to map all the Illumina reads to the scaffolds generated by 454 Newbler. The inter- and intra-scaffold gaps were filled by local assembly of Roche and Illumina reads. The gaps between contigs were closed by targeted PCR amplification and sequencing of the PCR products with BigDye terminator chemistry on an ABI 3730 capillary sequencer.

Plasmid DNA extracted from *E. coli* EcNDM1 was transformed into *E. coli* JM109, and a carbapenems resistant transformant carrying plasmid pEcNDM1-4 named JM109 (pEcNDM1-4) was recovered. Plasmid pEcNDM1-4 extracted from the *E. coli* JM109 (pEcNDM1-4) was used to construct a Tn5 transposon library according to the manufacturer's recommendations (Epicenter, Madison, WI) [22]. Each transposon clone was sequenced using the BigDye terminator chemistry on an ABI 3730 capillary sequencer, first with primer FP-V and primer RP-V to obtain unique sequences flanking the transposon and then by PCR walking sequencing.

The pEcNDM1-4 equivalent plasmid in NDM-1 negative strain *E. coli* EcNDMneg, named pEcNDMneg-4, was sequenced by targeted sequencing. Twenty pairs of overlapping primers were designed according to the sequences of plasmid pEcNDM1-4 (Table S1 in File S1) and used for the amplification of pEcNDMneg-4 as overlapping PCR products which were sequenced.

### Nucleotide sequences accession numbers

Sequences of the NDM-1 carrying plasmids were submitted to the GenBank database with accession numbers JX515588, JX469383 and JX515587 for pRpNDM1-1, pEcNDM1-4 and pEcNDMneg-4 respectively.

## Results

### Isolation of NDM-1 positive *R. planticola* and *E. coli*

**Figure 1:**
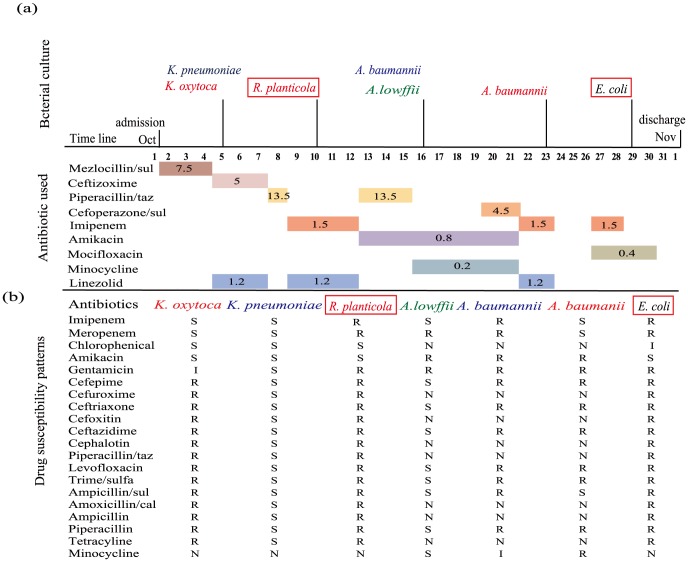
Time line of antibiotics treatment, microbiological investigation and isolation of NDM-1 positive *R. planticola* and *E. coli*. (**a**): Time line of events during the 34-day hospitalization of the patient. Bacterial species isolated at a particular time point are shown above the time line while antibiotics used and their durations are marked below the time line. The sources of strains are colour coded: red, from blood; blue, from sputum; green: from urine; black, from feces. Red boxed: NDM-1 positive bacteria. The number in bracket after a given antibiotic is dosage of the antibiotic used (g/day). (**b**): Antibiotics resistance profiles of bacteria isolated. Interpretation of results: S, susceptible; I, intermediate; R, resistant; N, not done.

Bacterial culture was attempted on four occasions to guide clinical care of the patient during hospitalization. From the blood culture of October 9^th^, 2010, a NDM-1 positive *R. planticola* strain, RpNDM1, was isolated. On October 29^th^, 2010, a NDM-1 positive *E. coli* strain, EcNDM1, was isolated from a fecal sample of the same patient. Additionally from the same fecal sample a multidrug resistant NDM-1 negative *E. coli* strain, EcNDMneg, was also isolated. We did not detect any NDM-1 positive bacteria from any other patients in the same ward. Medical records of the patient were reviewed for any evidence of association between administration of antibiotics and dissemination of *bla*_NDM-1_. Before the isolation of the NDM-1 positive *R. planticola*, only mezlocillin/sulbactum, ceftizoxime and linezolid were used and no carbapenems were used. However the NDM-1 positive *E. coli* was isolated after administration of imipenem, piperacillin/tazobactum, amikacin, minocycline and linezolid (Figure 1).

### Characterization of strains isolated from the patient

**Figure 2:**
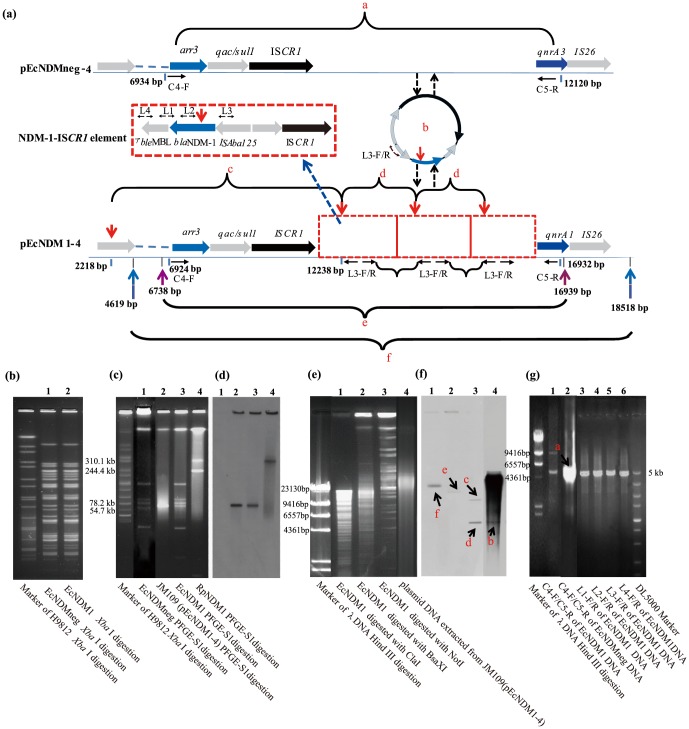
The characterization and mobilization model of the 4812-1-IS*CR 1* element. (**a**): Genetic context of plasmids pEcNDM1-4 and pEcNDMneg-4 and mobilization of the 4812 bp NDM-1-IS*CR1* element. Red boxes denote 4812 bp NDM-1-IS*CR1* element; Horizontal arrow denote the direction of primers; Red downward pointing arrows denote *Not*I restriction site; Blue upward pointing arrows denote *Cla*I restriction site; Purple upward pointing arrows *Bsa*X1 restriction site; Lowercase red letters denote corresponding fragments in (**f**) and (**g**). (**b**): PFGE of *Xba* I digested patterns of *E. coli* strains EcNDM1 and EcNDMneg. (**c**): PFGE of S1 digested plasmid profiles from *E. coli* EcNDM1, *R. planticola* RpNDM1 and transformant *E. coli* JM109 (pEcNDM1-4). (**d**): Southern blot hybridization of (**c**) with a *bla*_NDM-1_ probe. (**e**): PFGE of plasmid pEcNDM1-4 with or without restriction enzyme digestion stained with ethidium bromide. (**f**): Southern blot hybridization of (**e**) with a *bla*_NDM-1_ probe. Lowercase red letters denote fragments expected as depicted in (**a**). (**g**): PCR amplification for confirmation of the structure of the NDM-1-IS*CR1* element with primer pairs as shown in (**a**).

Both RpNDM1 and EcNDM1 were resistant to carbapenems, penicillins and cephalosporins and sensitive to amikacin, colistin sulphate and tigecycline. Additionally, EcNDM1 was also resistant to aztreonam, gentamicin and ciprofloxacin (Table 1). The two *E. coli* strains EcNDM1 and EcNDMneg had identical resistance patterns with the sole exception that the former was also resistant to carbapenems. PCR sequencing of the *bla*_NDM-1_ from both RpNDM1 and EcNDM1 revealed 100% identity with the published *bla*_NDM-1_ sequence [3]. PFGE-*Xba* I analysis of EcNDM1 and EcNDMneg showed identical PFGE patterns (Figure 2b), suggesting that the two strains had the same origin.

**Table 1:** Antimicrobial susceptibility patterns of *R. planticola* KpNDM1, *E. coli* EcNDMneg, *E. coli* EcNDM1-4 and their transformants.

Antibiotics	Minimum inhibitory concentration (mg/L)					
	*R. planticola*	*E. coli*	*E. coli*	*E. coli*	*E. coli*	*E. coli*
	RpNDM1	EcNDM1	EcNDMneg	EcNDMneg (4812)	JM109 (pEcNDM1-4)	JM109
TZP	>256	>256	16	32	>256	0.75
CTX	>256	>256	>256	>256	>256	0.25
ATM	0.094	32	32	32	0.064	0.064
GEN	4	16	16	16	0.125	0.094
AMK	3	6	6	6	1.5	0.5
CIP	0.75	>32	>32	>32	0.5	0.032
IMP	>32	>32	0.19	16	16	0.19
MEM	>32	>32	0.23	16	16	0.016
CST	0.5	0.75	0.5	0.5	0.125	0.125
TGC	0.75	0.38	0.38	0.38	0.38	0.25
MHT*	positive	positive	negative	positive	positive	negative

TZP: piperacillin/tazobactam; CTX: cefotaxime; ATM: aztreonam; GEN: gentamicin; AMK: amikacin; CIP: ciprofloxacin; IPM: imipenem; MEM: meropenem; CST: colistin; TGC: tigecycline.

MHT*: Modified hodge test was performed and interpreted according to 2011 CLSI recommendation.

### The *bla*_NDM-1_ location and plasmid analysis

PFGE of S1 digested plasmid DNA and Southern hybridization were performed to examine plasmid profiles and the *bla*_NDM-1_ location of *E. coli* EcNDM1, EcNDMneg and *R. planticola* RpNDM1. RpNDM1 had two large plasmids, designated as pRpNDM1-1 and pRpNDM1-2 of approximately 280 kb and 240 kb respectively and *bla*_NDM-1_ was found to be on the larger plasmid pRpNDM1-1 by Southern hybridization (Figure 2c and 2d). EcNDM1 had five plasmids ranging from 25 kb to 180 kb, which were named as pEcNDM1-1 to pEcNDM1-5 in descending order of their sizes. The *bla*_NDM-1_ was found to be on the 60 kb plasmid pEcNDM1-4 by Southern hybridization (Figure 2d). *E. coli* strain EcNDMneg shared an identical plasmid profile with EcNDM1. The plasmid pEcNDMneg-4 equivalent to plasmid pEcNDM1-4 in EcNDMneg was found to have no hybridization signal with the *bla*_NDM-1_ probe (Figure 2c and 2d).

Conjugation was performed with EcNDM1, EcNDMneg and RpNDM respectively as donor and *E. coli* J53 Az^r^ as recipient. Both pEcNDM1-4 and pEcNDMneg-4 were found to be a conjugative plasmid with a relatively high transfer frequency at approximately 1×10^−3^ per donor cell. However pRpNDM1-1 failed to transfer into *E. coli* J53 Az^r^ by conjugation.

### Sequence analysis of plasmids pRpNDM1-1, pEcNDM1-4 and pEcNDMneg-4

**Figure 3:**
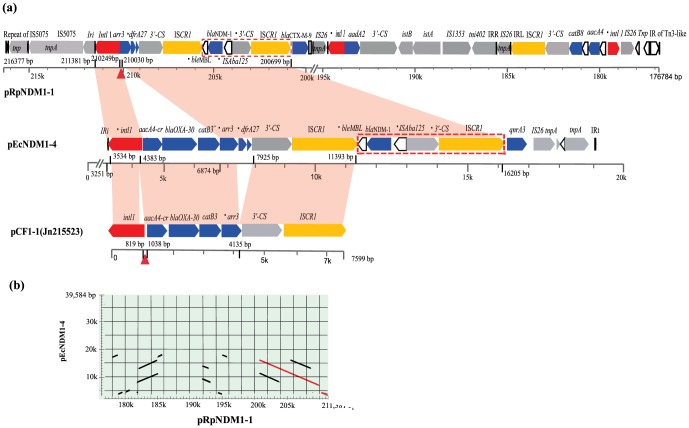
Schematic representation of the DNA sequences surrounding the *bla*_NDM-1_ in plasmid pEcNDM1-4, pRpNDM1-1 and homologous sequences in plasmid pCF1-1 to illustrate the relationship of the three plasmids. (**a**): Illustration of the adjacent genes of NDM-1-IS*CR1* of pRpNDM1-1, pEcNDM1-4 and homologous sequences in plasmid pCF1-1. Arrows represent ORFs and their direction of transcription; Filled triangles show the same 218 bp insertion in pCF1-1 and in pRpNDM1-1, compared with pEcNDM1-4; Open rectangle represent hypothetical proteins between *bla*_CTX-M-9_ and IS26 in plasmid pRpNDM1-1; Pink shades across the plasmids indicates more than 99% identity between sequences. (**b**): MUMer-based genomic display between pEcNDM1-4 and pRpNDM1-1. The corresponding homologous sequences represented in (**a**) were highlighted in red lines.

Plasmid pRpNDM1-1 was sequenced by whole genome sequencing of RpNDM1 using a combination of Roche 454 sequencing with 283 867 paired-end reads generated for a 18.48 -fold coverage and Illumina sequencing with 15 713 532 75-bases paired end reads for an approximately 285.7-fold coverage. Most of the Roche and Illumina reads were assembled into 252 contigs and 23 scaffolds. Gaps were closed and genome structure confirmed by targeted PCR and Sanger sequencing. The chromosome and the two plasmids of RpNDM1 were completely assembled. The *bla*_NDM-1_ carrying plasmid pRpNDM1-1 was 277 682 bp in size and belongs to the IncH group by sequence homology analysis [23], [24]. The plasmid encodes 261 coding sequences (CDSs), of which 136 encode proteins with homology to proteins of known functions and the remaining 125 (47.9%) encode hypothetical proteins. The *bla*_NDM-1_ was found within a 39 594 bp antibiotic resistance region, starting from a Tn3-like 38 bp invert repeat (IR) upstream of *merR* (encoding putative transcriptional regulator) at bp 176 784 to the end of the interrupted 38 bp IR of Tn5036 at bp 216 377 and was flanked by a 5 bp direct repeat, characteristic of Tn3-family transposons. Besides *bla*_NDM-1_, six antibiotic resistance genes, *aacA4* (resistance to aminoglycoside), *catB8* (resistance to chloramphenicol), *sul1* (resistance to sulphonamides), *aadA2* (resistance to aminoglycoside), *bla*_CTX-M-9_ (resistance to lactam antibiotics), *arr3* (resistance to rifampin) and a truncated *dfrA27* (resistance to trimethoprim) with a 113 bp deletion in the middle of the gene were also found in this region (Figure 3a).

pRpNDM1-1 was found to share 86% of its sequence by length with *Klebsiella oxytoca* strain E718 plasmid pKOX-R1 (GenBank accession number CP003684), showing three segments of co-linearity, although genetic arrangement of the segments was slightly different (Figure S2 in File S1). In comparison to pKOX-R1, pRpNDM1-1 carries an additional 32 395 bp antibiotic resistance region, starting from downstream of IS26 at position bp 177 631 to upstream of class 1 integron 5′CS at position bp 21 0027, carrying 7 antibiotic resistance genes including *bla*_NDM-1_. Interestingly, *Klebsiella oxytoca* strain E718, a NDM-1 carrying strain, isolated from Taiwan harbored another plasmid, pKOX_NDM1 in addition to pKOX-R1. The *bla*_NDM-1_ was found to be located on pKOX_NDM1, which had little similarity with pRpNDM1-1 except for a 100% identical segment of 1294 bp carrying 5′-IS*Aba125*-*bla*_NDM-1_-*ble*_MBL_-3′, which indicates that the NDM-1 acquisition by plasmid pRpNDM1-1 has a complex history (Figure S2 in File S1).

The plasmid pEcNDM1-4 was sequenced using the EZ-Tn5 transposition system. The genetic structure and the sequence of the plasmid were further confirmed by PCR and sequencing of the PCR products using 19 pairs of overlapping primers. The plasmid was 58 228 bp and was found to share a backbone of approximately 51 kb with plasmid pKp96 [25] at 99% DNA sequence identity including the complete array of genes for replication, plasmid transfer, partition and stabilization (Figure S1 in File S1). The backbone of pEcNDM1-4 was interspersed by four insertions. Insertion-1 (bp 4384 to bp 7924) was comprised of *bla*_OXA-30_ (resistance to lactam antibiotics), *catB3* (resistance to chlorophenical), a truncated *arr3* and a truncated *dfrA27*. The truncated *dfrA27* is unusual as it contained a 113 bp deletion in the middle of the gene and is identical to that found in plasmid pRpNDM1-1. Insertion-2 (bp 11 394 to bp 16 257) was comprised of a truncated *ble*_MBL_(resistance to bleomycin), *bla*_NDM-1_, a truncated IS*Aba125*, a truncated *qacE1/sul1* and an intact IS*CR1*. Insertion-3 (bp 17 091 to bp 17 909) was comprised of a mobile element IS26. Insertion-4 (bp 35 885 to bp 36 170) was comprised of a partial *fipA* encoding a fertility inhibition protein (Figure S1 in File S1).

The plasmid pEcNDMneg-4 was sequenced by targeted PCR sequencing using the 19 pairs of primers that were used to confirm the genetic structure of pEcNDM1-4 (Table S1 in File S1). Except for 2 pairs of primers, 17 pairs of the primers successfully amplified products and the sequences obtained from sequencing the PCR products were identical to that of pEcNDM1-4. We found that pEcNDMneg-4 was completely co-linear and identical to pEcNDM1-4 except for the region where the 2 pairs of primers failed. The gap was closed using new primers and the plasmid pEcNDMneg-4 was 53 416 bp and was 100% identical to plasmid pEcNDM1-4 except for a 4812 bp deletion which contained a truncated *ble*_MBL_, *bla*_NDM-1_, a truncated IS*Aba125*, the class 1 integron 3′CS (*qacE1/sul1*) and an IS*CR1* element. This 4812 bp NDM-1-IS*CR1* element is discussed in detail below.

### Comparison of the two *bla*_NDM-1_ carrying plasmids, pRpNDM1-1 and pEcNDM1-4

The two *bla*_NDM-1_ carrying plasmids, pRpNDM1-1 and pEcNDM1-4, were unrelated in plasmid backbone and genetic arrangement, except for a 9332 bp-segment between bp 6874 and bp 16 205 in pEcNDM1-4 and between bp 210 030 and bp 200 699 in pRpNDM1-1 which was 100% identical (Figure 3b). The 9332 bp-segment consisted of a truncated *arr3*, an unusually truncated *dfrA27*, 3′-CS, IS*CR1*, a truncated *ble*_MBL_, *bla*_NDM-1_, a truncated IS*Aba125* and a second copy of 3′-CS and an IS*CR1* with a truncted *qacE1*. This segment was located in a complex class 1 integron in both pRpNDM1-1 and pEcNDM1-4. In comparison to the complex class 1 integron in pRpNDM1-1, the complex class 1 integron in pEcNDM1-4 contained a 218 bp deletion in the 5′ CS and immediately downstream of the 5′ CS an additional gene cassette array *aacA4*-*bla*_OXA-30_-*catB3* which is prevalent in *Enterobacteriaceae* plasmids, such as plasmid pCF1-1 (Figure 3a).

### The discovery and characterization of the 4812 bp NDM-1-IS*CR1* element

When the plasmid DNA extracted from JM109 (pEcNDM1-4) was probed with the *bla*_NDM-1_ probe, a 5 kb band (Figure 2f, labelled b) hybridized with the *bla*_NDM-1_ probe at low intensity although this band cannot be seen on the agarose gel (Figure 2e), in addition to the heavy smear band that corresponds to the *bla*_NDM-1_ carrying 58 kb plasmid. The 5 kb band was unexpected, suggesting that a free form of a 5 kb DNA molecule carrying *bla*_NDM-1_ exists in the cell.

Using primer pair C4-F and C5-R designed to amplify the sequence between *arr3* and *qnrA3* (Figure 2a), two fragments of approximately 5 kb and 10 kb were amplified from pEcNDM1-4 (Figure 2g, Lane 1) and one fragment of approximately 5 kb was amplified from pEcNDMneg-4 (Figure 2g, Lane 2). The latter was much more intense, although similar amounts of templates were used for the 2 PCR amplifications. Cloning and sequencing of the 5 kb and 10 kb agarose gel purified fragments from the pEcNDM1-4 PCR amplification revealed that the 10 kb fragment corresponds to the region between *arr3* to *qnrA3* (see Figure 2a) and the 5 kb fragment corresponds to the same region but is devoid of the 4812 bp NDM-1-IS*CR1* element. We also sequenced the 5 kb PCR product from pEcNDMneg-4. The sequences of the two 5 kb fragments were 100% identical. These results suggest that the 4812 bp NDM-1-IS*CR1* element can be excised from the plasmid. The 5 kb band observed in the Southern hybridization of undigested plasmid DNA extract described above must be the 4812 bp NDM-1-IS*CR1* element, providing further evidence of the existence of a free form of the 4812 bp NDM-1-IS*CR1* element.

Four pairs of primers, L1F/R to L4F/R, facing diverging directions were designed to confirm the structure of the 4812 bp NDM-1-IS*CR1* element. An approximately 5 kb fragment was amplified from EcNDM1 using each of the four pairs of primers (Figure 2g). Sequencing of these PCR products showed that they all corresponded to the 4812 bp NDM-1-IS*CR1* element, albeit the sequences started from different positions as the primers anneal at different sites. These PCR experiments confirmed the presence of the 4812 bp NDM-1-IS*CR1* element. However the PCR results cannot determine whether the NDM-1-IS*CR1* element is in a circular form or in tandem multiple copies since the diverging primer pairs can amplify a product in both scenarios.

We then used restriction mapping to determine whether there is more than one copy of the NDM-1-IS*CR1* element on the plasmid. There is a *Not*I site at bp 12 238 located in the 4812 bp NDM-1-IS*CR1* element and a *Not*I site upstream at bp 2218 (Figure 2a). When the plasmid pEcNDM1-4 was digested by *Not*I and probed with the *bla*_NDM-1_ probe, a 10 kb hybridization band was observed as expected (Figure 2e and 2f). A 5 kb hybridization band was also observed and was approximately double of the intensity of the 10 kb hybridization band, suggesting that there were double copies of the 5 kb band relative to the 10 kb band. We then used 2 more restriction enzymes, *Cla*I and *Bsa*XI, to further determine whether there is a single copy or multiple copies of the NDM-1-IS*CR1* element. The *Cla*I (restriction sites flanking the *bla*_NDM-1_ gene at bp 4619 and bp 18 518) and *Bsa*XI (restriction sites flanking the *bla*_NDM-1_ gene at bp 6738 and bp 16 939) digestions were expected to generate 13 kb and 10 kb fragments respectively. However when plasmid pEcNDM1-4 was digested by *Cla*I and *Bsa*XI and probed with the *bla*_NDM-1_ probe, a 23 kb band and a 20 kb band were hybridized with the *bla*_NDM-1_ probe respectively, which is 10 kb larger than expected (Figure 2f). The extra 10 kb indicates that there are 2 more copies of the NDM-1-IS*CR1* element present in tandem. However this result is inconsistent with the C4-F/C5-R PCR results which only detected a 5 kb and a 10 kb band corresponding to 0 or 1 copy of the NDM-1-IS*CR1* element. The discrepancy can be explained by that the C4-F/C5-R PCR failed to amplify the 20 kb amplicon that contains 3 copies of the NDM-1-IS*CR1* element because of the length limit of the PCR amplification. This also explains the large difference in band intensity observed between pEcNDM1-4 and pEcNDMneg-4 in the C4-F/C5-R PCR described above. There must be a very small proportion of the pEcNDM1-4 carrying 0 or 1 copy of the NDM-1-IS*CR1* element with the majority carrying 3 copies. Therefore the pEcNDM1-4 size is expected to vary from 53 416 kb (0 copy of the NDM-1-IS*CR1* element) to 67 852 kb (3 copies of the NDM-1-IS*CR1* element), which may also partly explain the smear observed in Figure 2e and Figure 2f of the total plasmid extract. Note that the assembled pEcNDM1-4 sequence has only one copy of the NDM-1-IS*CR1* element with a size of 58 228 bp, due to the limitation of sequence assembly that cannot resolve the repeats.

Considering all of the results above, the 4812 bp NDM-1-IS*CR1* element exists in two forms in EcNDM1 as illustrated in Figure 2a. One was located on the plasmid predominantly in three tandem copies, while the other was an extra-plasmid and extra-chromosomal free form. The latter must have been released from the plasmid in small quantities during replication, which may have resulted in some plasmids containing aberrant copies of the 4812 bp NDM-1-IS*CR1* element.

### The 4812 bp NDM-1-IS*CR1* element was transferable by transformation to *E. coli* EcNDMneg

The transferability of the 4812 bp NDM1-IS*CR1* element was examined by using *E. coli* strain EcNDMneg as a host which is sensitive to imipenem. The 4812 bp NDM-1-IS*CR1* element was amplified by PCR using primer pair L5-F/R (Table S1 in File S1) and the PCR product was ligated by T4 DNA ligase to become circular. The PCR product was introduced into *E. coli* EcNDMneg by electroporation, and a carbapenem-resistant transformant named EcNDMneg (4812) was found. The susceptibility profile of EcNDMneg (4812) was identical to that of strain EcNDM1 (Table 1). Targeted PCR sequencing found that the 4812 bp NDM-1-IS*CR1* element precisely inserted downstream of the existing IS*CR1* in pEcNDM1-4 to recreate an identical gene arrangement to pEcNDM1-4.

## Discussion

This case of isolating two NDM-1 positive strains of different species, *R. planticola* and *E. coli* isolated from the same patient provided an interesting scenario of emergence of NDM-1 within the same patient *in vivo*. The NDM-1 positive *R. planticola* strain was isolated when only mezlocillin/sulbactum, ceftizoxime and linezolid were used, suggesting that the emerging of NDM-1 was not due to administration of carbapenem antibiotics in this case. However, the NDM-1 positive *E. coli* was isolated after administration of imipenem which may have allowed the selection of an *E. coli* strain that possessed NDM-1. The 9332 bp fragment carrying an unusually truncated *dfrA27* gene was shared by the two plasmids, suggesting an additional link between the two *bla*_NDM-1_ carrying plasmids pRpNDM-1 and pEcNDM1-4. The latter further harboured an additional gene cassette array, *aacA4-cr*-*bla*_OXA-30_-*catB3*, which is highly prevalent in *Enterobacteriaceae*, with a 218 bp deletion in the 5′-CS. This deletion would most likely have been generated when the gene cassette array was inserted in pEcNDM1-4. Therefore the likely process is that *E. coli* acquired *bla*_NDM-1_from *R. planticola* and additionally acquired the gene cassette array *aacA4*-*bla*_OXA-30_-*catB3* from another *Enterobacteriaceae* plasmid, such as pCF1-1. The alternative is acquisition of *bla*_NDM-1_ by *R. planticola* from *E. coli*, which is also possible but less likely since acquisition of a 218 bp region to undo the deletion in pRpNDM1-1 is less probable. There is also a possibility that *bla*_NDM-1_was gained from another source by one or both species independently.

IS*CR1* element was first observed in class 1 integrons In6 and In7 in 1993 [26]. Since then, IS*CR1* elements were shown to have a close association with trimethoprim, quinolone, aminoglycoside resistance genes and several β-lactamase genes. Two copies of IS*CR1* with a 3′CS upstream flanking different subtypes of *qnr* genes were also reported in China [27], France [17] and other countries [28]. *bla*_NDM-1_ has always been found to be carried on a structure consisting of a partial IS*Aba125* and a *ble*_MBL_ gene with *bla*_NDM-1_ sandwiched between them [3]. IS*CR1* elements adjacent to the conserved IS*Aba125*-*bla*_NDM-1_- *ble*_MBL_ structure have been reported [3], [16], [18]. However, this is the first time to report that *bla*_NDM-1_ was flanked by an IS*CR1* element on both sides. The conserved 1596 bp IS*Aba125*-*bla*_NDM-1_- *ble*_MBL_ region was flanked by a copy of the integron 3′CS *qacE1/sul* and IS*CR1* on both sides (Figure 3a) in the two NDM-1 carrying plasmids of different species *R. planticola* and *E. coli*.

IS*CR1* is a known means of mobilizing its adjacent sequence via rolling circle transposition. Partridge and Hall [14] and Toleman *et al.* [15] demonstrated that rolling circle replication of the IS*CR1* element can produce circular modules that include the IS*CR1* and its upstream adjacent antibiotic resistance genes. These circular modules can then be rescued by recombination between homologous fragments. The 4812 bp NDM-1-IS*CR1* element may be mobilized by rolling circle transposition. Our isolation of two nearly identical *E. coli* strains from the same fecal sample with only one being NDM-1 positive presented an interesting scenario for the acquisition of *bla*_NDM-1_ and provided a vehicle for experimental transfer of NDM-1 antibiotic resistance. Our *in vitro* transformation experiment using a synthesized circular 4812 bp NDM-1-IS*CR1*element showed that the element successfully transformed EcNDMneg containing plasmid pEcNDMneg-4 to a NDM-1 positive strain with the precise insertion of the element into the IS*CR1* downstream in pEcNDMneg-4 to create an identical gene arrangement to pEcNDM1-4, demonstrating its mobility via IS*CR1*. However, *in vitro* transformation is artificial. There are likely other mechanisms of transfer.

The advantage for the *bla*_NDM-1_ gene embedding in a class 1 integron IS*CR1* complex is that one of the most widely used mechanisms for the spread of antibiotics resistance across species is harnessed for NDM-1 dissemination. Therefore in addition to clonal expansion of a NDM-1 positive strain, NDM-1 can spread by multiple means including inter-strain spread within and between species through plasmids, transposons and the NDM-1-IS*CR1* element. Given that strains carrying class 1 integrons or IS*CR1* complex class1 integrons are common among bacteria [13], [15], [29], this is potentially a highly effective means for the spread of *bla*_NDM-1_.

In conclusion, *bla*_NDM-1_ was found to be embedded in an IS*CR1* complex class 1 integron, which is most likely responsible for the *bla*_NDM-1_ transfer between *R. planticola* and *E. coli* in a patient *in vivo*. The novel 4812 bp NDM-1-IS*CR1* element which can self excise to become a free form could provide a new vehicle for NDM-1 dissemination.

## Supporting Information

File S1 Figure S1, Figure S2 and Table S1.
(DOC)
