# Supplementary material for: Sequential Isolation in a Patient of Raoultella planticola and Escherichia coli Bearing a Novel ISCR1 Element Carrying bla NDM-1
Source: PLoS One. 2014 Mar 3;9(3):e89893. doi: 10.1371/journal.pone.0089893 (PMC3940617; doi:10.1371/journal.pone.0089893)
Supplement: File S1 — Figure S1, Figure S2 and Table S1. (DOC) [file pone.0089893.s001.doc]

**Supplements:**

**Table S1. Primers used in this study.**

| **Name** | **Primer sequences(5’-3’)** | **Position** | **Length (bp) ^#^** |
| --- | --- | --- | --- |
| NDM-F | caggcaacagccgaacga | 11483..11500 | 1 193 |
| NDM-R | cgttagattggcttacaccattaga | 12676..12652 |
|  |  |  |  |
| NDM-Southern-F | tcagcgcagcttgtcggcc | 11678..11696 | 560 |
| NDM-Southern-R | gcgtgctggtggtcgatac | 12237..12219 |
|  |  |  |  |
| L1-F | gaattcgagctgcaaaccgc | 11534..11553 | 4 812 liner |
| L1-R | ttcccctatcctgacctcga | 11533.. 11514 |
|  |  |  |  |
| L2-F | tcagcgcagcttgtcggccat | 11678..11698 | 4 783 |
| L2-R | tgccatcgcgcgatttcgat | 11649..11630 |
|  |  |  |  |
| L3-F | atgcacccgctcagcatcaat | 12411..12431 | 4 783 |
| L3-R | cagcaaatggaaactggcga | 12382..12363 |
|  |  |  |  |
| L4-F | ttcaccaatgccaccatggcat | 11445..11466 | 4 765 |
| L4-F | gctccgggtataggaagtat | 11398..11379 |
|  |  |  |  |
| L5-F | gacatctcacgtccacggtcat | 12874..12895 | 4 812 circle |
| L5-R | actgcgtaaaacgttgacct | 12915..12896 |
|  |  |  |  |
| C1-F | agcagtctaacgagcttacc | 35..54 | 2 983 |
| C1-R | tctacgcatcttcaggatgt | 3036..3017 |
|  |  |  |  |
| C2-F | ttcaggtcgtcgagtaaagt | 2804..2823 | 3 426 |
| C2-R | cttgcctttgaaggggctat | 6229..6210 |
|  |  |  |  |
| C3-F | tgtccgcacttacaggaaac | 5962..5981 | 1 367 |
| C3-R | agtcctccaacgaatccaac | 7328..7309 |
|  |  |  |  |
| C4-F | ggattcccatctctcatgat | 6924..6943 | 4 721 (0) * |
| C4-R | atcgcgcgatttcgatgtgaca | 11645..11624 |
|  |  |  |  |
| C5-F | aatgccaccatggcatcgag | 11451..11470 | 5 482 (0) * |
| C5-R | ggcagcactatgactcccaa | 16932..16913 |
|  |  |  |  |
| C6-F | ttcagcaagaggatttctca | 16305..16324 | 3 720 |
| C6-R | ggcgattgctgacagaattg | 20024..20005 |
|  |  |  |  |
| C7-F | tatctctacagcaagcgcca | 19755..19774 | 3 343 |
| C7-R | gcttgccgttgtattcttcc | 23097..23078 |
|  |  |  |  |
| C8-F | atggccgcgatagcgtttag | 22803..22822 | 3 424 |
| C8-R | attcgaaacatgcggtcgtc | 26226..26207 |
|  |  |  |  |
| C9-F | cgtaggaagctgaacaacgc | 25959..25978 | 3 380 |
| C9-R | attgcttcgctgggcgtcat | 29338..29319 |
|  |  |  |  |
| C10-F | cctactgagctggctaaaca | 29065..29046 | 3 122 |
| C10-R | aggttggtatcactcatggc | 32186..32167 |
|  |  |  |  |
| C11-F | agcagcgttatgtcaacccg | 31810..31829 | 2 379 |
| C11-R | ttaatggcctgcatggtcag | 34188..34169 |
|  |  |  |  |
| C12-F | tttcagcggcgtttatacgc | 33899..33918 | 3 291 |
| C12-R | atgctggcggagaatcatac | 37189..37170 |
|  |  |  |  |
| C13-F | cagtgaagtagctgattgtc | 36940..36959 | 3 427 |
| C13-R | tcgcgtgttgttcaacatag | 40366..40347 |
|  |  |  |  |
| C14-F | cagtgccggttgtcatgtta | 40047..40066 | 3 381 |
| C14-R | gacgagctggaatcactcca | 43427..43408 |
|  |  |  |  |
| C15-F | gagtgagcgcgacatttgat | 43203..43222 | 3 069 |
| C15-R | gccagtacatcctctacttc | 46271..46252 |
|  |  |  |  |
| C16-F | tggtgcgtcaaacgtagaag | 46050..46069 | 3 312 |
| C16-R | gcgctttaaggagtacacca | 49361..49342 |
|  |  |  |  |
| C17-F | tatggcactctgacgccata | 49129..49148 | 3 900 |
| C17-R | cgtgttgagtgtgcttcttg | 53028..53009 |
|  |  |  |  |
| C18-F | gacgaatcatgataggaggc | 52691..52710 | 3 630 |
| C18-R | tgacaacatctgacggcgca | 56320..56301 |
|  |  |  |  |
| C19-F | gatcatgctggccgatttca | 56134..56153 | 2 447 |
| C19-R | atcggctgccagcctctgtta | 352..332 |
|  |  |  |  |
| C4-F | ggattcccatctctcatgat | 6924..6943 | 10009 (5197)* |
| C5-R | ggcagcactatgactcccaa | 16932..16913 |

#, the length of PCR products was predicted plasmid pEcNDM1-4as template.

*, The numbers in the bracket indicate the length of PCR product amplified from the plasmid pEcNDMneg-4.

**Figure S1. Genetic arrangement of plasmids pEcNDM1-4, pEcNDMneg-4 and pKp96 to illustrate the relations of the three plasmids.**

**(a):** MUMer-based genomic display between pEcNDM1-4**,** and pKp96. The corresponding homologous sequences were highlighted in red lines.

**(b):** Illustration of the three plasmids pEcNDM1-4, pEcNDMneg-4 and pKp96. Arrows represent ORFs and their direction of transcription; Red filled triangles show 218 bp fragment insertion in pKp96 compared to pEcNDM1-4 and pEcNDMneg-4; Blue filled triangles show 54 bp fragment insertion in pEcNDM1-4 and pEcNDMneg-4 compared to pKp96; pink and yellow shades across the plasmids show regions of more than 99% and more than 94% DNA sequence identity.

**Figure S2. Comparison of plasmid pRpNDM1-1 and the plasmids pKOX_NDM1 and pKOX-R1 from NDM-1 positive *Klebsiella oxytoca* E718.**

**(a):** MUMer-based genomic display between pRpNDM1-1 and pKOX_NDM1. The 1294 bp homologous segment carrying *bla*_NDN-1_ is highlighted in red lines. Dash lines indicate the 1294 bp boundaries of 100% identical sequences between pRpNDM1-1 and pKOX_NDM1.

**(b):** MUMer-based genomic display between pRpNDM1-1 and pKOX-R1. The insertion segment carrying *bla*_NDN-1_ is indicated by dash lines in plasmid pRpNDM1-1 and illustrated in **(c)**.

**(c):** Illustration of the genetic arrangement of *bla*_NDN-1_ flanking sequences in plasmids pRpNDM1-1, pKOX_NDM1 and pKOX-R1. Dashed lines indicate the boundaries of nearly identical sequences between pRpNDM1-1 and the other two plasmids. Arrows represent ORFs and their direction of transcription; homologous genes between plasmids are colour coded: gray; mobility related genes; red: antimicrobial resistance genes; yellow: 3’CS and IS*CR1* genes; blue: other function genes; and white: hypothetical proteins.

**Figure S1**


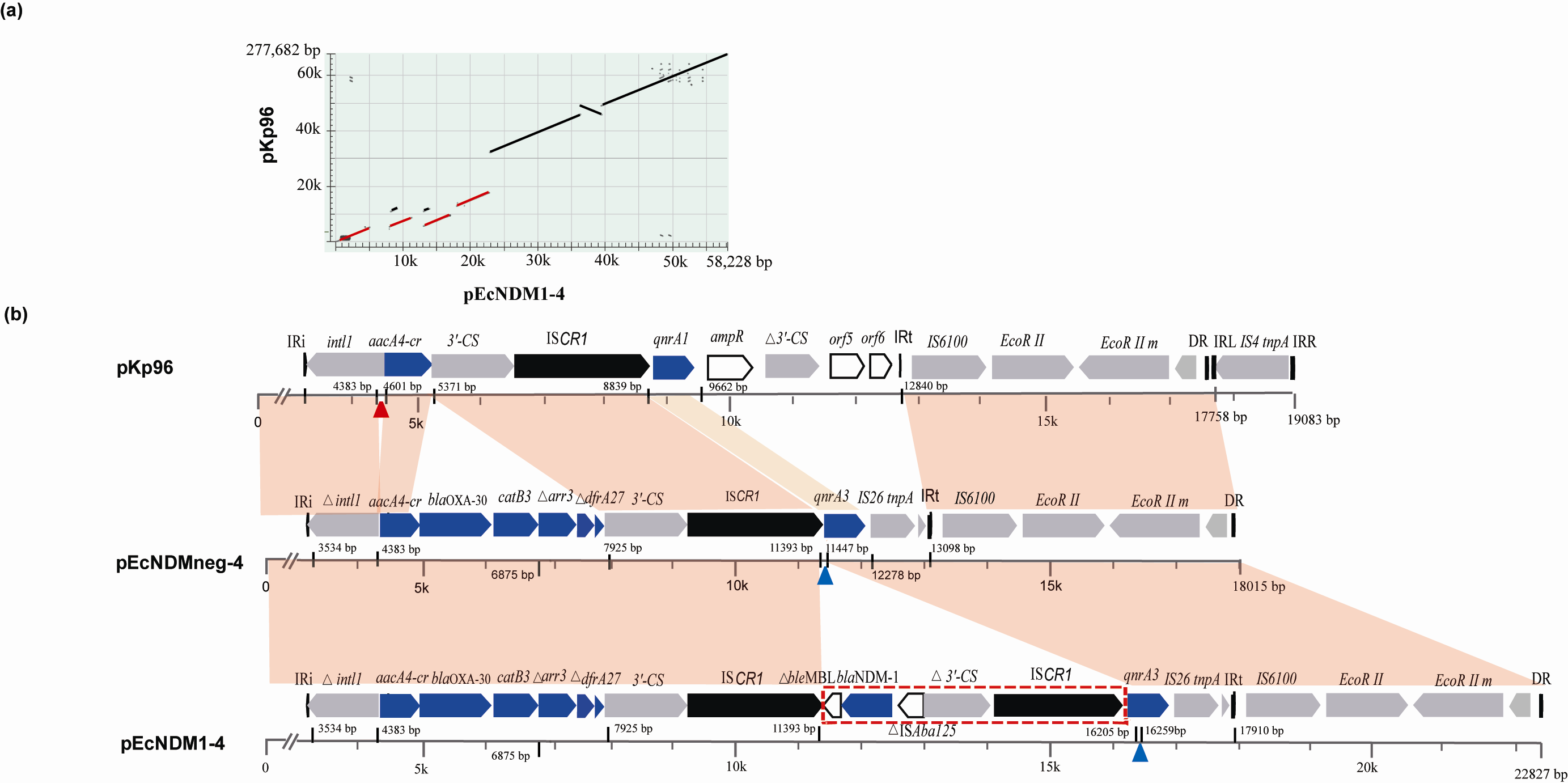


**Figure S2**
